# Morphology of immature stages of the black fig fly *Silbaadipata* (Diptera, Lonchaeidae)

**DOI:** 10.3897/BDJ.12.e137798

**Published:** 2024-11-21

**Authors:** Miguel Á Juárez Maya, Óscar Morales Galván, Jorge M Valdez Carrasco, Daniel Illescas Salvador, José M Gutierrez Ruelas, Carlos P Illescas Riquelme

**Affiliations:** 1 Colegio de Postgraduados, Campus Montecillo, Posgrado en Fitosanidad-Entomología y Acarología, Texcoco, Estado de México, Mexico Colegio de Postgraduados, Campus Montecillo, Posgrado en Fitosanidad-Entomología y Acarología Texcoco, Estado de México Mexico; 2 Universidad Autónoma Chapingo, Departamento de Parasitología, Texcoco, Estado de México, Mexico Universidad Autónoma Chapingo, Departamento de Parasitología Texcoco, Estado de México Mexico; 3 SADER-SENASICA-DGSV, Dirección de Protección Fitosanitaria, Ciudad de México, Mexico SADER-SENASICA-DGSV, Dirección de Protección Fitosanitaria Ciudad de México Mexico; 4 CONAHCYT/ Centro de Investigación en Química Aplicada, Departamento de Biociencias y Agrotecnología., Saltillo, Coahuila., Mexico CONAHCYT/ Centro de Investigación en Química Aplicada, Departamento de Biociencias y Agrotecnología. Saltillo, Coahuila. Mexico

**Keywords:** fruit flies, *Ficuscarica*, quarantine pests

## Abstract

The black fig fly *Silbaadipata* is an exotic and invasive pest of figs in several producing areas of Mexico. The larvae of this species feed on the internal tissue of the syconium, causing rot and premature drop. In addition to *S.adipata*, other species of fruit flies can be associated with figs in Mexico. Therefore the recognition of their immature stages is useful for plant health inspection procedures and timely management decisions. This study aimed to describe the egg, larva (L3) and puparium stages of *S.adipata*, provide photographic material for their recognition and discuss the most important external morphological characteristics to differentiate them from *Zaprionusindianus* and *Anastrephaludens* and discriminated amongst other species of Diptera of the Drosophilidae and Tephritidae families associated with figs.

## Introduction

The fig tree (*Ficuscarica* L.) is a deciduous and evergreen tree belonging to the family Moraceae, composed of numerous varieties with great genetic diversity (Ben-Abdallah et al. 2023, Ramadan 2023). This plant is native to the Middle East. It has been dispersed by human action to different regions around the world, mainly due to its commercial value and high adaptability to different environments, soils, climates and altitudes (Akbar 2020, Ben-Temessek et al. 2023).

The main product of this plant is its syconia, commonly called figs. These are sold fresh or dehydrated for human consumption, their positive effects on health or the manufacture of various by-products depending on the geographical area (Ben-Temessek et al. 2023). According to data from SIAP (2024), Mexico has a planted area of 2,168 ha, with a production of 12,489 t, in 19 Federative Entities, with a production value around 16 million USD, with the State of Morelos being the main producer. The Mexican fig production is destined for the export market, mainly to the USA and Canada (SENASICA 2024, SE 2024).

The incidence of pests and diseases is one of the major problems in the production and marketing of figs. Within this complex of organisms, some species of fruit flies are the most important due to their negative impact on yield, increase in production costs and the trade restrictions imposed by fruit-importing countries to limit their entry and protect their phytosanitary status (Aluja and Mangan 2008).

Fruit flies (Diptera) lay eggs on healthy fruits and their larvae feed on them, making them unmarketable. Fruit flies associated with figs around the world belong to the families Lonchaeidae, Drosophilidae, Tephritidae (Silvestri 1917a, Gonçalves et al. 2008, MacGowan et al. 2012, Mifsud et al. 2012, Radonjić et al. 2019, Arimoto et al. 2020, Akşit and Çakmak 2022, Singh et al. 2022) and Chloropidae (Silvestri 1917b, Nartshuk 2011); however, the diversity and importance of the species vary depending on the geographic region.

The black fig fly, *Silbaadipata* McAlpine (Diptera, Lonchaeidae), is a monophagous and invasive species native to the Mediterranean and the Middle East (Britt et al. 2022). Females lay eggs in the ostiole and the larvae subsequently feed on the internal tissues of the fig, causing its premature drop (Abbes et al. 2021; Suppl. material 1). In Mexico, it was initially reported in commercial fig orchards in Ayala, Morelos (NAPPO 2020) and later in backyard fig trees in the State of Mexico (Bautista-Martínez et al. 2021). Currently, this pest has spread to several areas of Mexico and is the most devastating in terms of production (Paniagua-Jasso et al. 2024). This has meant that fresh Mexican figs for export must undergo a radiation quarantine treatment in order to access the US market (APHIS 2021).

Other flies (Diptera, Drosophilidae) associated with figs in Mexico are the African fig fly *Zaprionusindianus* (Gupta), the spot-winged fly *Drosophilasuzukii* (Matsumara) and the vinegar fly *Drosophilamelanogaster* (L.) (Bautista-Martínez et al. 2017). The larvae of these three species can develop in the syconia. However, *Z.indianus* is the only one of this family considered a primary pest of *F.carica* by attacking mature figs; the last two are secondary or opportunistic.

On the other hand, fig is mentioned as a host for several species of Tephritidae (Diptera, Brachycera) such as *Anastrephaludens* (Loew) and *Anastrepha* spp. by the Mexican Official Standards NOM-025-FITO-1995 and NOM-075-FITO-1997 (SAGARPA 1998, SAGARPA 1999). However, in our opinion, this parasite-host relationship is for prevention purposes, since no study has been conducted to confirm *F.carica* as a host for species of this genus. The lack of comprehensive studies confirming this relationship underscores the need for further research in this area. Given that multiple groups of fruit flies are connected with figs in Mexico, it is crucial to accurately identify the species in their immature stages. This is because they can be discovered in the field within figs or at various points in the product logistics chain. The present work describes the immature stages of *S.adipate*, indicating the most important parts for its recognition and mentioning characteristics that distinguish it from species of Drosophilidae and Tephritidae.

## Materials and methods

**Immature stages of *Silbaadipata*.** Developing figs vr. Black Mission with signs of irregular maturity, stem rot and emergence holes were collected in commercial orchards located in the Municipalities of Tlayecac (18°45'38.466"N, 98°53'50.5932"W, 1,316 m a.s.l.), Jaloxtoc (18°44'19.2182"N, 98°55'2.77"W, 1,277 m a.s.l.) and Tetela del Volcán, (18°50'20.7"N, 98°45'16.5"W, 1,481 m a.s.l.), belonging to the State of Morelos, México in September 2021, June 2022 and March 2023. The collected material was placed in plastic containers and transferred to the insect taxonomy laboratory of the Departamento de Parasitología Agrícola de la Universidad Autónoma Chapingo, Texcoco, State of Mexico.

From the syconia in the containers, some newly-emerged larvae (L3) were selected, these were boiled in water for 30 seconds, then preserved in 70% alcohol. The rest of the emerged larvae were allowed to continue their cycle to obtain pupae and adults. The males were identified from the chaetotaxy of the thorax and genitalia, with the keys and illustrations of McAlpine (1956), McAlpine and Steyskal (1982), McAlpine (1987), MacGowan and Freidberg (2008) and MacGowan and Rotheray (2021). To obtain eggs, *S.adipata* adults were confined in entomological cages (25 × 25 × 25 cm). Adults were supplied with tap water in cotton and a sugar-based diet with hydrolyed protein was constantly provided with immature figs until the females laid eggs.

**Morphological study of immature stages.** Twenty-six larvae (L3) preserved in alcohol were selected and photographs were taken of their bodies; anterior and posterior spiracles; mouth-hooks; oral ridges and anal lobes with their respective dimensions. To illustrate the cephalopharyngeal skeleton and posterior spiracles, the body's first anterior third and posterior third were separated and immersed in 10% potassium hydroxide (KOH) solution at 80°C for 20 minutes to macerate the tissue. They were then washed with running water and 70% and 100% alcohol. Finally, they were immersed in xylene and then mounted on a slide glass with Canadian balsam to take the image.

The microscopy, image processing and analysis work were done at the Insect Morphology Laboratory of the Colegio de Postgraduados, Campus Montecillo, Texcoco, State of Mexico. The photographs were taken with a Carl Zeiss SteREO Discovery V20 microscope (Germany), with a Canon 2000D digital camera (Japan). Darktable 4.8.1, ZereneStacker 1.04 and GIMP 2.10.34 programmes were used to capture and manage the images. The measurements were obtained with the ImageJ 1.53t image analyser.

The description of immature states was made, based on the schemes of McAlpine (1961), McAlpine (1987), Frias et al. (2006), MacGowan et al. (2012), MacGowan and Rotheray (2021) and Rodriguez et al. (2022). To make comparative observations with *Z.indianus* and *A.ludens*, eggs, larvae and puparium of this species were obtained from the Agricultural Entomology collection of the Department of Entomology and Acarology of the Colegio de Postgraduados Campus Montecillo.

## Results and Discussion

**Figure 1.**
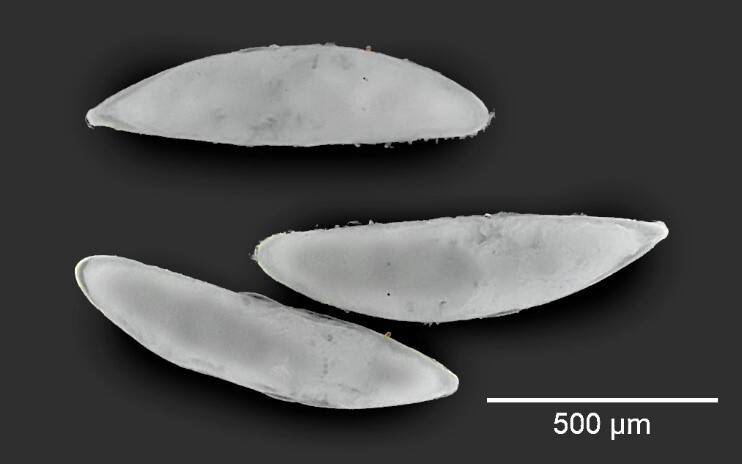
*Silbaadipata* eggs.

*Silbaadipata* eggs are about 1.2 mm long, whitish, translucent, smooth, elongated and almost straight (Fig. 1).

**Figure 2.**
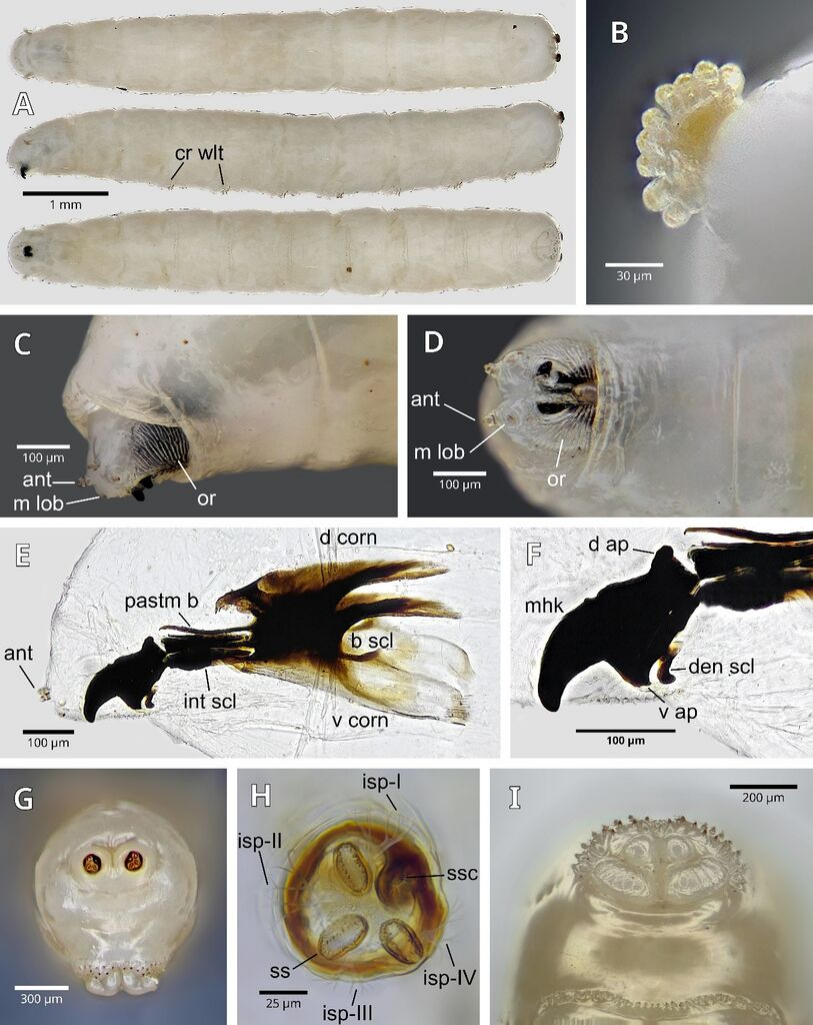
*Silbaadipata* larva. **A** Lateral, ventral and dorsal views of habitus; **B** Anterior spiracle; **C** Pseudocefalon in lateral view; **D** Pseudocefalon in ventral view; **E** Larval cephaloskeleton; **F** Mouth-hook; **G** Caudal segment; **H** Left posterior spiracle; **I** Anal lobes. Abbreviations: ant – antenna; b scl – basal sclerite; cr wlt – creeping welts; d corn – dorsal cornu; den scl – dental sclerite; int scl – intermediate sclerite; m lob - maxillary lobes; mhk – mouthhook; pastm b – parastomal bar; v corn – ventral cornu; or – oral ridges; d ap – dorsal apodeme; v ap – ventral apodeme; b mhk - mouth-hook base; isp – interspiracular processes; ss – spiracular slit; ssc – stigmatic scar.

Larvae are yellowish-white in colour, slender and cylindrical. The ventral abdominal area has creeping welts with some barely evident spines (Fig. 2A). Average length is 6.12 mm, height is 1.044 mm and width is 1.004 mm in abdominal segment V. Anterior spiracles (Fig. 2B) have 8 to 9 digits or respiratory lobes, with a semi-elliptical arrangement and projecting forwards. The oral ridges are especially difficult to observe due to the translucent integument of this area and the retractile nature of the pseudocephalon (Fig. 2C and D). These can vary between specimens in terms of number, size and bifurcations. Antennae and maxillary lobes are on the front part of the pseudocephalon (Fig. 2C and D)

Cephaloskeleton (Fig. 2E) with mouth-hook with rectangular-based, very sclerotized, curved, smooth and non-bifurcated mouth-hooks, with a notch at their upper margin, between the dorsal apodeme and the tip of the hook. Rounded dorsal apodeme and ventral apodeme with a pointed end directed backwards. Dental sclerite, wide and curved, located behind the ventral apodeme (Fig. 2F). Intermediate sclerite sclerotized, wide, bar-shaped, parallel to the thinner parastomal bar. No accessory oral sclerites are observed. Dorsal cornu highly sclerotized. Ventral cornu slightly sclerotized. Basal sclerite shows black sclerotization, mostly confined to the anterior end of the basal sclerite and the dorsal cornu.

Posterior spiracles (Fig. 2G) are located above the mid-line of the caudal segment; they are elevated, short and very sclerotized, characteristic of Lonchaeidae. The plates have three spiracular slits almost straight, longer than wide. The upper and lower slits are almost perpendicular to the central spiracular slit. In the spiracular plate (Fig. 2H), there is a stigmatic scar on the inner margin and four hyaline interspiracular processes that alternate between the spiracular slit and the stigmatic scar. They comprise a main trunk that bifurcates and ends in 8 to 10 points. It has bifid anal lobes (Fig. 2I), with the posterior part smaller than the anterior and has two to three rows of thick, sclerotizedmicrospines.

**Figure 3.**
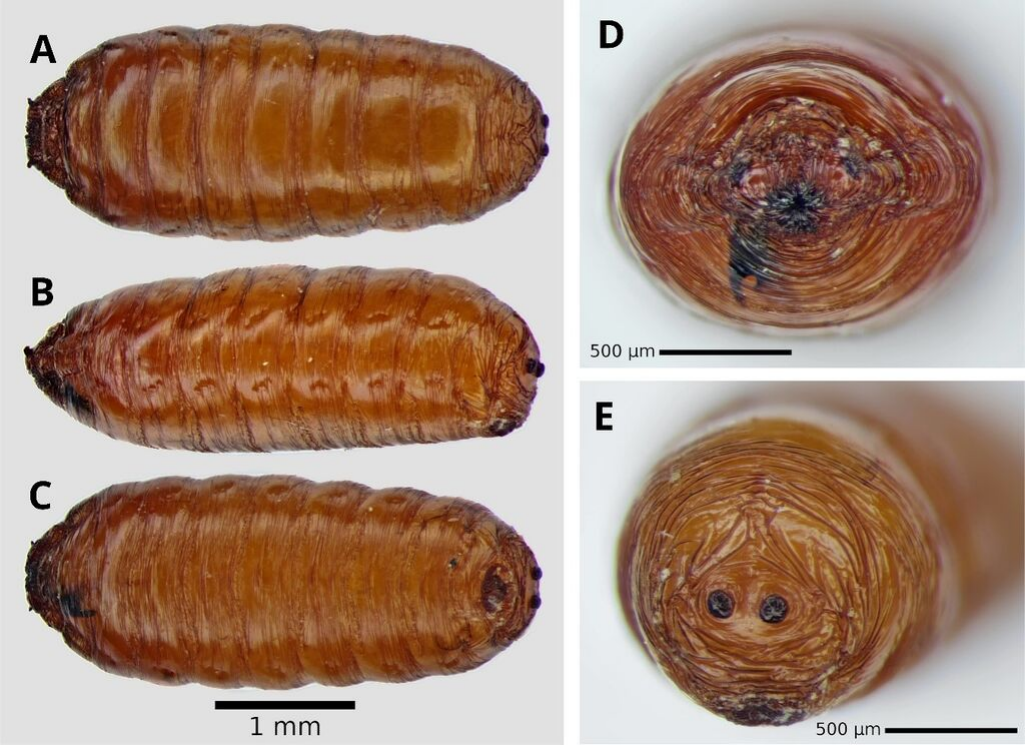
*Silbaadipata* puparium. **A** Dorsal view; **B** lateral view; **C** Ventral view; **D** Anterior; **E** Posterior.

Puparium of *S.adipata* is reddish-brown, cylindrical and has 11 well-defined segments. The posterior part is slightly narrower than the anterior. It has several transverse striations mostly visible at the anterior, posterior and ventral ends. The vestiges of the anterior and posterior spiracles of the larva are preserved (Fig. 3).

The information provided in the present study is useful for recognising the immature stages of the black fig fly and distinguishing it from other groups of fruit flies, Drosophilidae and Tephritidae, related to figs in Mexico and other countries in the Americas.

*Silbaadipata* eggs can be easily differentiated from those of Drosophilidae species that lay eggs in the ostiole or within the syconium, such as *Z.indianus*, *D.susukii* and *D.melanogaster*, since they have different numbers of respiratory filaments (4, 2 and 2, respectively) (Matavelli et al. 2013, Bautista-Martínez et al. 2017), which are absent in *S.adipata*. On the other hand, *Anastrepha* eggs are generally longer than those of *S.adipata* and have a curved shape resembling a banana (Figueiredo et al. 2013).

The general morphology of *S.adipata* larvae presents characteristics that allow them to be easily differentiated from the Drosophilidae species associated with fig. The larvae of *Zaprionusindianus* and *Drosophila* spp. are smaller and translucent; their anterior spiracles have a general stalk that ends in several long filamentous processes capable of retracting into the body and their posterior spiracles are small, tubular, slightly sclerotic and project towards the posterior part (Teskey 1981, Matavelli et al. 2013, Van-Timmeren et al. 2017).

In general, *Anastrepha* larvae are larger and more robust than *S.adipata* larvae; however, they have some similarities since both are phylogenetically related within the superfamily Tephritoidea (Han and Ro 2016) and could be confused in early instars. In particular, *A.ludens* differs from *S.adipata* because it has more digits in the anterior spiracle (+12). Its posterior spiracles are not prominent; its spiracular openings are almost linear and parallel to each other. Its oral ridges (11-17) are well defined and it also has fine microspines surrounding the anal lobes (Carroll and Wharton 1989, Hernández-Ortiz et al. 2020, FAO 2021, Rodriguez et al. 2022). The cephalopharyngeal skeleton of *Anastrepha* larvae and several species of Tephritidae present a well-formed and sclerotized anterior sclerite (Frias et al. 2006, Steck and Ekesi 2015, Rodriguez et al. 2022), which is absent in *S.adipata*.

Dimensions and shapes of the mouth-hooks and cephaloskeleton of *S.adipata* are similar to those of *Silbalashker* (Diptera, Lonchaeidae), another phytophagous species associated with syconia of *Ficuscarica* in India (MacGowan et al. 2012). Further information will be needed to determine whether these attributes are sufficient to separate it from *Neosilba* species associated with figs in South America (Uchôa and Nicácio 2010, Nicácio and Uchôa 2011, Raga et al. 2015, Sousa et al. 2021).

The puparium of *S.adipata* retains the shape of the short and sclerotized posterior spiracles, typical of several groups of Lonchaeidae. In *Z.indianus*, as in *Drosophila* spp., the pupae have tubular projections with filaments corresponding to the anterior spiracles (Matavelli et al. 2013). The puparium of *Anastrephaludens* is larger and oval in shape (SENASICA 2017).

Silvestri (1917a) made a very detailed description of *S.adipata* (referred as *Lonchaeaaristella*), where he illustrated diagrams of the morphology of the development stages. Description of the immature stages coincides with what was observed in the present work; there is no other report where this description is made. Using optical microscopy, it was possible to illustrate the anterior spiracle, the cephaloskeleton, posterior spiracles, interspiracular processes and anal lobes of *S.adipata*, important structures for its characterisation and separation from other dipteran larvae associated with figs, characteristics that can be useful during phytosanitary inspections at any port of entry.

## Supplementary Material

13289811-F438-5549-8BA5-02E8AF3822F8 10.3897/BDJ.12.e137798.suppl1 Supplementary material 1 Damage caused by Silbaadipata in figs Data type Image Brief description 1. Female of *S.adipata* laying eggs in the ostiole; 2. Figs with internal rot and galleries; 3. Larvae of *S.adipata* inside the figs. File: oo_1137016.tif https://binary.pensoft.net/file/1137016 Carlos Patricio Illescas Riquelme

## References

[B12064767] Abbes Khaled, Hafsi Abir, Harbi Ahlem, Mars Messaoud, Chermiti Brahim (2021). The black fig fly *Silbaadipata* (Diptera: Lonchaeidae) as an emerging pest in Tunisia: preliminary data on geographic distribution, bioecology and damage. Phytoparasitica.

[B12064811] Akbar Shahid (2020). *Ficuscarica* L. (Moraceae). Handbook of 200 Medicinal Plants.

[B12066472] Akşit T, Çakmak I, Zeynel D (2022). *Ficuscarica*: production, cultivation and uses.

[B12064820] Aluja Martín, Mangan Robert L. (2008). Fruit Fly (Diptera: Tephritidae) host status determination: Critical conceptual, methodological, and regulatory considerations. Annual Review of Entomology.

[B12064829] APHIS (2021). Import restrictions on fig fruit from Mexico.. https://www.aphis.usda.gov/sites/default/files/da-2021-18.pdf.

[B12064837] Arimoto Kôichi, MacGowan Iain, Su Zhi-Hui (2020). New data on lance flies (Diptera, Lonchaeidae) associated with figs (Moraceae, *Ficus* spp.) in Japan and Taiwan, with descriptions of two new species of the genus *Silba* Macquart. Journal of Asia-Pacific Entomology.

[B12064846] Bautista-Martínez Néstor, Illescas-Riquelme Carlos Patricio, López-Bautista Everardo, Velazquez-Moreno Lucia Jairoth, García-Ávila Clemente de Jesús (2017). Presence of Drosophilidae (Diptera: Ephydroidea) flies associated with fig fruits in Morelos, Mexico. Florida Entomologist.

[B12066529] Bautista-Martínez Néstor, Meraz-Álvarez Ricardo, Valdez-Carrasco Jorge Manuel, López-Bautista Everardo (2021). Black fig fly, *Silbaadipata* McAlpine, in backyards of the State of Mexico. Southwestern Entomologist.

[B12065134] Ben-Abdallah R, Othmani I, Lagha A, Fattouch S, Ramadan M F (2023). Fig (*Ficuscarica*): Production, processing, and properties.

[B12065179] Ben-Temessek M, Talbi W, Chrifa H, Fattouch S, Ramadan M F (2023). Fig (*Ficuscarica*): Production, processing, and properties..

[B12064897] Britt Kadie E, Gordon Phoebe E, Faber Ben A, Rios Sonia I, Wilson Houston (2022). First report of black fig fly, *Silbaadipata* (Diptera: Lonchaeidae), in the United States. Journal of Integrated Pest Management.

[B12064907] Carroll Lynn E., Wharton Robert A. (1989). Morphology of the immature of *Anastrephaludens* (Diptera: Tephritidae). Annals of the Entomological Society of America.

[B12064924] FAO ISPM (International Standards for Phytosanitary Measures) 27. Diagnostic protocols for regulated pests. DP 9: Genus *Anastrepha* Schiner. FAO. https://www.ippc.int/en/publications/81502/.

[B12064932] Figueiredo J. V. A., Perondini André L. P., Ruggiro Eliana M., Prezotto Leandro F., Selivon Denise (2013). External eggshell morphology of *Anastrepha* fruit flies (Diptera: Tephritidae). Acta Zoologica.

[B12064942] Frias D, Hernández-Ortiz V, Vaccaro N C, Bartolucci A. F, Salles L A (2006). Comparative morphology of immature stages of some frugivorous species of fruit flies (Diptera: Tephritidae). Israel Journal of Entomology.

[B12064952] Gonçalves M. A., Andrade L., Almeida L., Pica M. C. (2008). Study of *Ceratitiscapitata* and *Lonchaeiaaristella* on fig trees. Acta Horticulturae.

[B12064961] Han Ho-Yeon, Ro Kyung-Eui (2016). Molecular phylogeny of the superfamily Tephritoidea (Insecta: Diptera) reanalysed based on expanded taxon sampling and sequence data. Journal of Zoological Systematics and Evolutionary Research.

[B12066580] Hernández-Ortiz V, Hernández-López M, Steck G J, Montoya P, Toledo J., Hernández E. (2020). Moscas de la fruta: Fundamentos y procedimientos para su manejo.

[B12064970] MacGowan I, Freidberg A (2008). The Lonchaeidae (Diptera) of Israel, with descriptions of three new species. Israel Journal of Entomology.

[B12064979] MacGowan I, Razak N, Rotheray G. E., Ahmad I (2012). A new species of fig-feeding Lonchaeidae (Diptera: Schizophora) from India and a checklist for the family in the Indian sub-continent. Zootaxa.

[B12066538] MacGowan I, Rotheray G E, Kirk-Spriggs A H, Sinclair B J (2021). Manual of afrotropical diptera. Brachycera–Cyclorrhapha, excluding Calyptratae.

[B12065033] Matavelli Cristiane, Zara Fernando José, Zuben Claudio José Von (2013). Post-embryonic development in *Zaprionusindianus* (Diptera: Drosophilidae). Annals of the Entomological Society of America.

[B12065015] McAlpine J. F. (1956). Old world Lonchaeids of the genus *Silba* Macquart (= *Carpolonchaea* Bezzi), with descriptions of six new species (Diptera: Lonchaeidae). The Canadian Entomologist.

[B12065024] McAlpine J. F. (1961). A new species of *Dasiops* (Diptera: Lonchaeidae) injurious to apricots. The Canadian Entomologist.

[B12065096] McAlpine J. F., Steyskal G. C. (1982). A revision of *Neosilba* McAlpine with a key to the world genera of Lonchaeidae (Diptera). The Canadian Entomologist.

[B12066624] McAlpine J F, McAlpine J F, Peterson B V, Shewell G E, Teskey H J, Vockerothm J R, Wood D M (1987). Manual of nearctic diptera.

[B12066452] Mifsud D, Falzon A, Malumphy C, De Lillo E, Vovlas N, Porcelli F (2012). On some arthropods associated with *Ficus* species (Moraceae) in the Maltese Islands.. Bulletin of the Entomological Society of Malta.

[B12244937] NAPPO Detection of black fig fly (*Silba adipata McAlpine*) in the municipality of Ayala, State of Morelos. https://www.pestalerts.org/nappo/official-pest-reports/926/.

[B12065042] Nartshuk E P (2011). Chloropidae from southern Sardinia (Diptera: Cyclorrhapha, Acalyptratae). Conservazione Habitat Invertebrati.

[B12065052] Nicácio José, Uchôa Manoel A. (2011). Diversity of frugivorous flies (Diptera: Tephritidae and Lonchaeidae) and their relationship with host plants (Angiospermae) in Environments of South Pantanal Region, Brazil. Florida Entomologist.

[B12244945] Paniagua-Jasso Eduardo, Tejeda-Reyes Manuel Alejandro, Martínez-Castillo Ana Mabel, Figueroa-de la Rosa José Isaac, García-Banderas Diana Vely, Palma-Castillo Luis Jesús, Illescas-Riquelme Carlos Patricio, Pineda-Guillermo Samuel (2024). Bioecological Parameters of the Black Fig Fly, *Silbaadipata* (Diptera: Lonchaeidae), Collected from Fig Crops in Mexico. Insects.

[B12066463] Radonjić Sanja, Hrnčić Snježana, Perović Tatjana (2019). Overview of fruit flies important for fruit production on the Montenegro seacoast. BASE.

[B12066593] Raga A, de Souza-Filho M. F., Strikis P. C., Marangoni Monte S. M N. (2015). Lance fly (Diptera: Lonchaeidae) host plants in the State of São Paulo, Southeast Brazil.. Entomotropica.

[B12065150] Ramadan M F, Ramadan M F (2023). Fig (*Ficuscarica*): Production, Processing, and Properties.

[B12065070] Rodriguez Erick J., Steck Gary J., Moore Matthew R., Norrbom Allen L., Diaz Jessica, Somma Louis A., Ruiz-Arce Raul, Sutton Bruce D., Nolazco Norma, Muller Alies, Branham Marc A. (2022). Exceptional larval morphology of nine species of the *Anastrephamucronota* species group (Diptera, Tephritidae). ZooKeys.

[B12067420] SAGARPA (1998). Norma Oficial Mexicana NOM-075-FITO-1997. https://www.dof.gob.mx/nota_detalle.php?codigo=4875258&fecha=23/04/1998#gsc.tab=0.

[B12067428] SAGARPA (1999). Norma Oficial Mexicana NOM-023-FITO-1995. https://www.gob.mx/agricultura/documentos/norma-oficial-mexicana-nom-023-fito-1995.

[B12064916] SE Higos frescos o secos. Intercambio comercial de México.. https://www.economia.gob.mx/datamexico/es/profile/product/figs-fresh-or-dried.

[B12066602] SENASICA (2017). Ficha técnica mosca mexicana de la fruta (*Anastrepha ludens* Loew). https://www.gob.mx/cms/uploads/attachment/file/249395/Anastrepha_ludens_Loew.pdf.

[B12066435] SENASICA Huertos de higo registrados para exportación a los Estados Unidos de América. https://www.gob.mx/senasica/documentos/huertos-de-higo-registrados-para-exportacion-a-los-estados-unidos-de-america?idiom=es.

[B12066427] SIAP Servicio de Información Agroalimentaria y Pesquera. Acciones y Programas Cierre de la producción agrícola. https://nube.siap.gob.mx/cierreagricola/.

[B12066443] Silvestri F (1917). Sulla *Lonchaeaaristella* Beck. (Diptera: Lonchaeidae) dannosa alle infiorescenze e fruttescenze del Caprifico e del Fico. Bolletino di Laboratorio Zoologia Generale e Agraria della R. Scuola Superiore d’ Agricoltura in Portici.

[B12066516] Silvestri F (1917). Descrizione di una Specie di *Oscinosoma* (Diptera: Chloropidae) osservato in fruttescenze di caprifico.. Bollettino di Laboratorio Zoologia Generale e Agraria della R. Scuola Superiore d'Agricoltura in Portici.

[B12066497] Singh S, Li Z, Zhang Y, Grieshop MJ, Giliomee J, Massimino-Cocuzza GE, Sandhu RK, Sarkhosh A, Yavari A, Ferguson L (2022). The fig: Botany, production and uses.

[B12065086] Sousa Ester Marques de, Louzeiro Léo Rodrigo Ferreira, Strikis Pedro Carlos, Souza-Filho Miguel Francisco, Raga Adalton (2021). Host plants and distribution records of lance flies (Diptera: Lonchaeidae) in São Paulo State, Brazil. EntomoBrasilis.

[B12066668] Steck Gary J., Ekesi Sunday (2015). Description of third instar larvae of *Ceratitisfasciventris*, *C.anonae*, *C.rosa* (FAR complex) and *C.capitata* (Diptera, Tephritidae). ZooKeys.

[B12066554] Teskey HJ, McAlpine J F, Peterson B V, Shewell G E, J Teskey H, R Vockeroth J, Wood D M (1981). Manual of Neartic Diptera.

[B12065115] Uchôa Manoel A, Nicácio José (2010). New records of neotropical fruit flies (Tephritidae), lance flies (Lonchaeidae) (Diptera: Tephritoidea), and their host plants in the South Pantanal and adjacent areas, Brazil. Annals of the Entomological Society of America.

[B12066569] Van-Timmeren Steven, Diepenbrock Lauren M, Bertone Matthew A, Burrack Hannah J, Isaacs Rufus (2017). A filter method for improved monitoring of *Drosophilasuzukii* (Diptera: Drosophilidae) larvae in fruit. Journal of Integrated Pest Management.

