# Supplementary figures and images for: Morphology of immature stages of the black fig fly Silbaadipata (Diptera, Lonchaeidae)
Source: Biodivers Data J. 2024 Nov 21;12:e137798. doi: 10.3897/BDJ.12.e137798 (PMC11605294; doi:10.3897/BDJ.12.e137798)

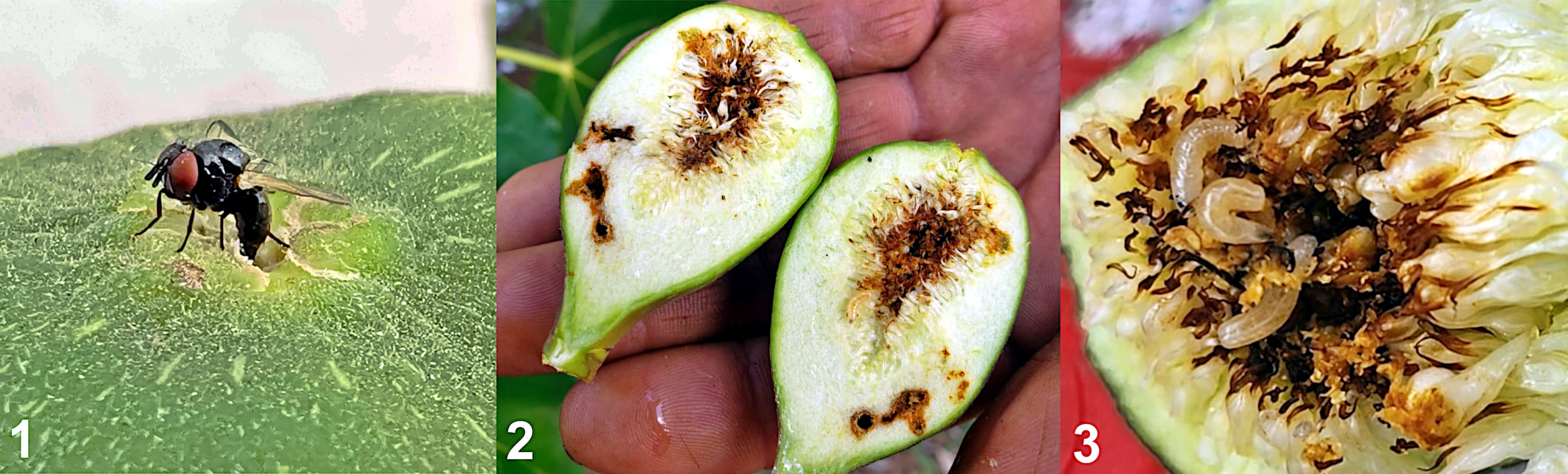

Supplement: Supplementary material 1 — Damage caused by Silbaadipata in figs [file bdj-12-e137798-s001.tif]
